# Calf health from birth to weaning. I. General aspects of disease prevention

**DOI:** 10.1186/2046-0481-64-10

**Published:** 2011-09-16

**Authors:** Ingrid Lorenz, John F Mee, Bernadette Earley, Simon J More

**Affiliations:** 1 Herd Health and Animal Husbandry, UCD School of Agriculture, Food Science and Veterinary Medicine, University College Dublin, Belfield, Dublin 4, Ireland; 2 Animal and Grassland Research and Innovation Centre, Teagasc Moorepark, Fermoy, Co. Cork, Ireland; 3 Animal and Bioscience Research Department, Teagasc, Animal and Grassland Research and Innovation Centre, Grange, Dunsany, Co. Meath, Ireland; 4 Centre for Veterinary Epidemiology and Risk Analysis, UCD School of Agriculture, Food Science and Veterinary Medicine, University College Dublin, Belfield, Dublin 4, Ireland

**Keywords:** Calf health, Prevention, Periparturient management, Colostrum, Dairy calf nutrition

## Abstract

Calfhood diseases have a major impact on the economic viability of cattle operations. This is the first in a three part review series on calf health from birth to weaning, focusing on preventive measures. The review considers both pre- and periparturient management factors influencing calf health, colostrum management in beef and dairy calves and further nutrition and weaning in dairy calves.

## Introduction

Calfhood diseases have a major impact on the economic viability of cattle operations, due to the direct costs of calf losses and treatment and the long term effects on performance [1]. Calf health was prioritised as one of the most important animal health issues facing the Irish livestock industry in a recent expert Policy Delphi study conducted on behalf of Animal Health Ireland (AHI) [2]. AHI was founded in 2009 as an industry-led, not-for-profit partnership between livestock farmers, processors, service providers and government, with the goal to improve the profitability, sustainability and competitiveness of Irish livestock farmers and related industries through superior animal health.

As part of ongoing AHI work, a group of experts was commissioned to provide evidence-based advice on calf health and disease management to Irish farmers, agricultural advisers and veterinary practitioners. As an initial step, a three-part review series on calf health from birth to weaning has been generated, specifically to provide a scientific evidence base for the development of advisory tools on calf health, and to identify gaps in current knowledge to be filled with targeted research. Even though the envisaged output will be specific for Irish husbandry systems, the scope of the reviews should make them useful for the same purpose elsewhere. The reviews cover both suckler and dairy calf management. However, due to the differences in the nature of these systems, some topics will deal mainly or exclusively with either dairy or suckler calves. This first part highlights issues relating to disease prevention in calves, with emphasis on the periparturient period, calving management, care of the newborn, colostrum management and further nutrition and weaning. The second and third parts focus on the management of diarrhoea in pre-weaned calves [3] and disease prevention and management with particular reference to calf pneumonia [4].

## Preparturient management factors influencing calf viability and health

Perinatal mortality is a problem in all eutherian species but particularly so in Holstein-Friesian-dominated dairy industries internationally [5]. Modifying preparturient management to improve calf viability and health is best achieved through implementation of simple protocols which document the correct strategies to be followed at the herd level and the correct procedures to be carried out at the individual animal level [6]. In addition, decisions taken earlier in the production cycle can influence calf viability, for example, choice of sire and sire breed, particularly beef breeds, use of sexed semen, age and weight at service in heifers, vaccination of the dam and nutrient intake in early pregnancy.

### Nutritional management in the last trimester

During the last trimester, adequate energy and protein should be provided whilst avoiding overfeeding in heifers to prevent foetal oversize, excess adipose deposition in the birth canal and resultant dystocia [7]. Preventing excess body condition score (BCS) in heifers prior to calving (target BCS of 2.75-3.0; scale 1-5) also has a significant beneficial effect on both the duration of parturition and incidence of perinatal mortality [8,9]. In contrast, cows losing excessive BCS may be carrying twins and should be dried off early, fed to maintain body condition and monitored for obstetrical complications at calving. In addition, placing beef heifers and cows on a straw diet prepartum to prevent potential dystocia can lower the immune status of both their colostrum and of their calves [10]. Where the basal diet consists of home-grown forage, commonly in beef suckler herds, this may necessitate additional supplementation of micronutrients to ensure adequate foetal nutrition [11]. In dairy herds, reducing the dietary cation anion difference (DCAD) in the transition period has been shown to affect a linear decrease in the incidence of milk fever [12] and hence can reduce the risk of slow calvings and compromised perinates. Where congenital joint laxity and dwarfism (CJLD) has been diagnosed, typically in beef suckler calves [13], dilution of the silage-only diet with alternative forages or grains is recommended.

### Pharmacological induction of parturition

If oversized calves are a problem based on previous experience, induction of parturition using dexamethasone at term can be used to deliver foetuses alive without any increase in dystocia [14]. Where induction of parturition has traditionally been practised in dairy cattle, there has been increased loss of calves, retained placenta and reduced milk production. This is, however, associated with early induction, not induction at term [15,16].

## Calving management factors influencing calf viability and health

The tenets of good calving management to improve calf viability and health are provision of a suitable maternity site, adequate but not intrusive calving supervision, correct obstetrical techniques and judicious utilisation of veterinary assistance. The importance of these factors was highlighted by a large-scale study of parturient problems in Friesian heifers which concluded that the primary determinant of whether a herd had high or low perinatal mortality was the quality of calving management [17].

### Maternity facilities

The design and availability of specialised maternity accommodation can have a significant bearing on calving outcomes, as reviewed recently by Mee [18]. In addition, maternity facilities can significantly impact calf health. Dairy calves born in maternity pens are less likely to develop diarrhoea than those born in non-maternity facilities (loose housing or stanchions) [19]. Individual (vs. group) maternity pens have been associated with increased calf plasma immunoglobulin concentration and reduced risk of enteric and respiratory disease in most [20-22] but not all studies [23]. Irrespective of the type of maternity facility, early removal of the calf (before standing) has been recommended to reduce calfhood morbidity and mortality on dairy farms in the USA [24].

The principal function of a maternity site is to simulate as closely as possible natural calving conditions as is the norm for extensively managed beef suckler cows. To simulate natural calving conditions for intensively managed dairy cows they should be moved to maternity accommodation prior to the onset of calving though studies comparing this with moving once calving has commenced have not been published. Counter intuitively, moving pregnant dairy cows and heifers later in the calving process when the placenta or fetal hooves are visible can reduce the odds of perinatal mortality compared to moving them earlier when mucus only is visible [25]. These results suggest that it is less detrimental to move animals which have already commenced calving (stage two) than it is to move animals which are about to start calving (stage one). It is likely that environmental disturbance, such as moving an animal, may cause psychogenic uterine atony if initiated in stage one of calving, but may only cause a temporary decrease in uterine motility if initiated in stage two. However, this strategy requires 24 h monitoring of the 'close-up group' with approximately hourly checks and it is not clear whether this policy may interrupt the calving process and lead to more calving problems than if these animals were not moved or were moved before stage one commenced.

### Calving supervision

Good calving supervision involves being present to assist during stage two of calving or to call for veterinary assistance, if required but not intervening unnecessarily. The day and time of calving is best predicted from altered behaviour such as increased frequency of rising and lying down, pawing with the forefeet and urinating and pelvic ligament relaxation [26]. Whilst there have been some interesting recent research developments in prediction of [27] and altering of the onset of calving in dairy cows [28] these have not yet been widely applied commercially. Lack of supervision can lead to perinatal death due to prolonged calving with resultant anoxia [29] or acidosis, which can predispose neonates to failure of passive transfer of colostral immunoglobulins [30]. The relevance of good calving supervision is highlighted by a survey of cow-calf operations in the US which showed that the majority of calvings did not take place in specialised maternity units and the majority of producers left heifers and cows calving for too long [31]. Intervention is generally recommended if the second stage of calving exceeds 2 h [32]. Tocolytic agents, such as clenbuterol, have been used successfully to both postpone night calvings and manage dystocia, but are not available in all jurisdictions. Various calving alarms have been developed to alert farmers to the time of calving such as biosensors that monitor postural behaviour, intravaginal or reticular temperature, vaginal mucus electrical resistance, myometrial contractions or tail elevation, but none are widely used commercially.

### Obstetrical technique

Training of farm staff with protocols for various obstetrical problems should be part of the role of modern veterinary practitioners in the transfer of technical knowledge [33], as although almost a third of calvings are assisted, less than 3% are attended by veterinarians [34]. Farmers with good obstetrical technique can prevent iatrogenic traumatic lesions, a major cause of perinatal mortality, particularly now that mechanical traction is commonly employed at calving. For example, recent research has shown that alternate limb traction should be applied until both elbows have entered the pelvis and simultaneous traction should then be applied to reduce the risk of trauma to the calf [35].

## Care of the newborn calf to prevent poor viability and ill-health

The emergency medicine concept of the 'golden hour' can be applied to at-risk newborn calves. This term refers to the principle of rapid intervention to prevent subsequent sequelae. High risk calves can be identified (a) before birth by the predicted likelihood of suffering from dystocia; (b) during birth by large forelimbs, swollen tongue, cyanosed muzzle and gums; or (c) after birth by apnoea or dyspnoea, lateral recumbence, flaccid musculature and poor pedal and suck reflexes. The triage approach to paediatric care of the at-risk bovine perinate in the first hour of life involves etho-physical assessment, resuscitation as necessary, umbilical antisepsis and colostrum feeding.

### Assessment of newborn calf vitality

The vigour of the newborn calf can be assessed immediately after calving by monitoring individual indicators (responsiveness to exogenous stimuli, muscle tone, sucking reflex, time to head lift and time to first standing) or a combination of indicators in a calf vigour score [36]. A calf should normally lift its head, attain sternal recumbency and attempt to stand and to stand spontaneously, on average, 3, 5, 20 and 60-90 minutes after birth, respectively [37,38].

### Calf resuscitation

Immediately after birth, calves suffering from mild fetal asphyxia should be hypothermally stimulated by pouring cold water over the head then suspended upside-down for up to a minute [39,40]. Once a patent airway has been established, the at-risk calf should be placed in sternal recumbence [39]. Mechanical ventilation should be implemented in cases which do not respond to these first aid measures [41]. While the clinical benefits of some pharmacological stimulants in newborn calves are equivocal, doxapram has recently been shown to be beneficial in cases of fetal asphyxia [42]. Buffer solutions containing sodium bicarbonate have safely been used recently to improve the acid-base status in acidotic perinatal calves [43]. Oxygen therapy for calf resuscitation is possible, even though not widely practised on commercial dairy or beef farms. A positive effect of this measure on perinatal survival has only been proven in cases of respiratory distress syndrome in calves born immature [44].

### Umbilical care

Prevention of omphalitis or 'navel ill' is based on good maternity pen hygiene, reducing calf residency time in unhygienic calving pens, ensuring adequate early intake of good quality colostrum and navel antisepsis [45]. In a recent review of navel care in perinates, Mee [29] concluded that producers should avoid possibly harmful cord application procedures and concentrate on maternity pen hygiene and calf immunity. In herds with serious navel-ill problems, producers should improve maternity pen hygiene, institute immediate and repeated cord dipping with chlorhexidine [46], removal of the calf immediately after birth to a clean calf pen, hand-feeding colostrum and regular checking for navel ill with metaphylactic parenteral antimicrobial therapy based on veterinary advice as necessary.

## Colostrum management

Due to the structure of the bovine placenta, the calf is born without protective immunoglobulins (Ig) and therefore depends on the successful passive transfer of maternal Ig from colostrum. Multiple studies have shown that failure of passive transfer (FPT, serum IgG < 10 g/L [47]) markedly increases morbidity and mortality in dairy and in beef calves (e.g. [10,48-52]. Besides immunoglobulins, colostrum provides a variety of other important ingredients like cytokines and growth factors as well as a superior nutritional value compared with whole milk [47].

In general, adequate passive transfer is subject of the quality of colostrum, the calf's ability to absorb Ig and the volume ingested.

The quality of colostrum in beef breeds is generally better than in dairy breeds and with average values in most studies well above 100 g/L good enough to provide adequate passive transfer, as long as supervision is provided to ensure colostral intake [10,52-54].

Lactation number, breed of cow and length of the non-lactating period (if less than 3 weeks) influence volume and Ig concentration of colostrum in dairy cows [55-57]. Mean colostral IgG concentrations of 68.5 g/L in Holstein cows were recently reported, whereby 32% of cows had poor colostrum quality (< 50 g/L) if milked within 1 h after calving. Pluriparous cows had higher IgG concentrations than primiparous cows in some, but not all studies [10,58-60]. Colostral IgG concentration decreases by 3.7% during each subsequent hour post calving; therefore, time of first milking is the most crucial factor regarding colostrum quality that the producer can influence [61]. The benefit of testing of colostrum quality on farm with commercially available hydrometers is controversial [47]. It appears to give good results only if the cut-points are adjusted for the specific device used [58]. More reliable results can be achieved using refractometry [62]. Pooling of colostrum lowers quality due to dilution and is also discouraged for reasons of biosecurity, e.g. transmission of *Mycobacterium avium *subsp. *paratuberculosis *[47].

Vaccination of the dam as a measure to increase specific antibody levels in colostrum will be discussed in the second part of this review [3].

The ability of the neonate to absorb IgG starts to decline progressively after 4 to 6 h and ceases after 24 h from birth [63,64]. Therefore, the earlier a calf is fed/suckles after birth, the greater the level of Ig absorption. Continuous feeding of smaller amounts of colostrum throughout the first two weeks of life has been associated with reduced diarrhoea in dairy calves, most probably due to local effects in the intestines [65].

It is currently recommended that normal sized dairy calves (Holstein-Friesian) are given either 3 L of good quality colostrum within 2 h of birth by oesophageal tube [59] or at least 3 L within 4 h and a total of 4 L within 12 h from birth by nipple feeding [60]. The amount of colostrum that calves drink voluntarily does not change within the first 4 h after birth, so that there is no benefit in delaying first feeding [60]. Feeding colostrum by stomach tube ensures successful passive transfer if high volumes are given [66,67]. However, if smaller volumes are given and the amount of immunoglobins administered is marginal it should be fed by nipple bottle, since the absorption of immunoglobulins in this situation is superior to that of stomach tubed calves [67]. In the US feeding colostrum by oesophageal feeder is used as a routine measure in about 14% of dairy heifer calves [68]. In Europe the discussion surrounding force-feeding colostrum is somewhat controversial, and further complicated by the fact that animal welfare legislation in some countries prohibits force feeding of animals except for medical reasons [69].

Suckling as a means of colostrum intake is associated with a higher risk of FPT compared with supervised feeding [69,70].

Bacterial contamination of colostrum occurs frequently on many dairies, with two associated concerns; a risk of transfer of infection and decreased absorption of IgG in the intestines. Total bacterial count should not exceed 100,000 colony forming units (cfu)/mL and faecal coliforms should be below 10,000 cfu/mL [24]. In practice, these goals can be achieved by means of hygienic harvesting, avoidance of bacterial contamination, as well as immediate refrigeration or freezing of surplus colostrum [24]. Routine pasteurisation methods (as recommended for whole milk) lead to reduced IgG concentrations [71,72] and increased viscosity [73]. Heat treatment at 60°C for 30 min reduces bacterial count, preserves IgG concentration and increases the apparent efficiency of absorption of IgG [74].

Colostrum replacement products (CR) are available for use if maternal colostrum is not available or is not given for biosecurity reasons. The efficacy of whey protein concentrate (WPC), used as a colostrum substitute and administered as a single feeding to dairy calves, was poor in preventing neonatal morbidity and mortality compared with a single feeding of pooled colostrums [75]. Studies evaluating the efficacy of commercial CR to prevent FPT in calves have produced very mixed and often unacceptable results [76]. Smith and Foster [77] concluded that simply examining the mass of IgG provided by the CR is not an adequate measure or predictor of product efficacy.

Frozen colostrum can be stored at -18 to -25°C for at least a year without changing its quality. Slow thawing at temperatures below 50°C does not affect colostrum quality, while temperatures above 50°C cause colostral proteins, including immunoglobulins, to denature [78,79].

Direct tests for measurement of IgG concentrations are reliable, but laboratory-based and relatively expensive. The accuracy of many indirect tests (sodium sulphite turbidity, zinc sulphate turbidity test, γ-glutamyl transferase (GGT) activity, whole-blood glutaraldehyde coagulation test) has been questioned [80,81]. The measurement of serum total protein by refractometer is the most reliable test for herd screening, based on a review by Weaver et al. [81]. A serum protein concentration of 52 g/L was found to be equivalent to 10 g/L serum IgG and is suggested as test threshold for healthy calves up to an age of 8 days [80].

## Further nutrition and weaning of the dairy calf

Traditionally, dairy calves have been fed milk or milk replacer to an amount of approximately 10% of the calf's body weight (BW) per day [82]. This level of nutrition ('restricted feeding') allows only for maintenance requirements and minimal weight gain under thermo-neutral conditions [83]. Restricted feeding was introduced to encourage calves to eat concentrates as early as possible and thus to minimise costs for relatively expensive liquid feeds. After the first 3 weeks of life, starter concentrate intake increases and the calves start to grow rapidly [84]. It has been known for a long time that calves can grow a lot faster if they are supplied with more nutrients [85,86]. However, worldwide interest in early calf nutrition has only recently been heightened, based on research from Diaz et al. [87] and Jasper and Weary [82], among others. Calves suckling their dam or otherwise fed ad libitum ingest about 20% of body weight (BW) per day and reach up to 1 kg of daily weight gain [86,88]. Furthermore, high volumes of milk or milk replacer fed to young calves do not cause diarrhoea; therefore, nutritional diarrhoea is a consequence of either inadequate quality of the liquid feed or of management failures (e.g. [82,87,89,90]).

Starter concentrate intake is negligible in the first 3 weeks of life. Therefore, calves on restricted feeding regimes are at most only able to achieve 20-30% of their biologically normal growth [91]. It is well established that under-nutrition in humans impairs the immune response [92]. In calves, a higher plane of nutrition improves immune function [93] and also lowers mortality and the incidence of diarrhoea and pneumonia [94-96].

A report on calf welfare [97] states that animal welfare is poor if average growth is reduced substantially, for example by 50%. Data are insufficient at this time to determine the overall economic benefit of feeding systems that allow normal biological growth. An intermediate volume of milk (approximately 15% of BW) is sufficient to allow calves to reach over 50% of their growth capacity under moderate weather conditions [83]. Also, these amounts of liquid feed can be provided in systems with twice a day feeding without exceeding the abomasal capacity.

The choice of liquid feed usually depends on availability and producer preferences. Feeding of non-saleable ('waste') milk is recognised as a risk factor for the transmission of infectious pathogens and it should therefore be pasteurised [95,98]. Feeding milk containing antibiotic residues from treated cows increases the risk of development of antibiotic resistance [99]. Milk replacers are lower in energy content than whole milk and vary widely in composition and quality. Products containing non-milk proteins are not suitable for very young calves [100]. Since the protein requirements increase rapidly with increased growth rates, products with increased concentration of crude protein (25-27%) should be used in programmes seeking normal or near-normal biological growth [101].

Most commonly, dairy calves are provided with liquid feed twice daily. No difference in calf performance between once or twice daily feeding was found when calves were raised on restricted feeding systems [102,103]. However, once-daily feeding will present problems if calves are fed for normal or near-normal biological growth in the first weeks of life: the volume of liquid feed provided would pose a high risk of overloading the abomasum if given in one feed [97]. On Irish dairy farms, Gleeson et al. [104] found no significant advantages in labour input either during feeding or in overall calf care between once-daily, twice-a-day or ad libitum feeding systems. Currently, relevant European legislation (laying down minimum standards for the protection of calves) demands that calves are fed at least twice daily (Council Directive 2008/119/EC, 2008). Consistent with the European Convention for the protection of animals kept for farming purposes (Appendix C: Special provisions for calves, 1993), this can only refer to feedings of milk or milk replacer in the young calf, since calves are totally dependent on liquid feed for at least the first three weeks of live.

Independent of the feeding system concentrates and water should be provided to calves at all times to enhance development of ruminal digestion. The amount of milk fed can then be reduced to 10% of BW at 3 weeks of age without any known negative impact [96,105]. Consumption of concentrates enables the development of ruminal epithelium necessary for the calf to digest solid feed. Additional forage feeding has no added value in calves bedded on clean straw that receive a calf starter concentrate with adequate coarseness (approximately 2,000 μm) [106]. Calves can be weaned once they consistently consume 1 kg of concentrates per day. This level of intake can usually be reached at an age of 5 to 6 weeks if access to palatable starter and water is available *ad libitum *[79]. To assure constant growth rates, weaning should preferably be introduced gradually with a decrease of volumes of liquid feed provided over a period of some days [107,108].

## Conclusions

There are a broad range of preventive measures that are fundamental to optimal calf health during the period from birth to weaning. An emphasis on prevention is critical, limiting the need for subsequent intervention, particularly with the management of diseases of the gastrointestinal and respiratory systems. This review highlights preventive measures from birth to weaning, as well as the preceding periparturient period.

## Conflict of interest statement

None of the authors of this paper has a financial or personal relationship with other people or organisations that could inappropriately influence or bias the content of the paper. The Technical Working Group includes employees of Pfizer Inc. (CC) and Volac Ireland (LG); these companies played no role in the design, development or journal submission of this review series.

## Authors' contributions

IL, JM and BE drafted the manuscript and compiled the literature. All authors made substantial inputs to the review, critically discussed the progressing manuscript and approved the final manuscript.
